# Microbial metabolites and their influence on the tumor microenvironment

**DOI:** 10.3389/fimmu.2025.1675677

**Published:** 2025-09-18

**Authors:** Huanglin Duan, Baisheng Xu, Peiyue Luo, Tao Chen, Jun Zou

**Affiliations:** ^1^ Department of Urology, The First People's Hospital of Xiushui, Jiujiang, Jiangxi, China; ^2^ The First Clinical College, Gannan Medical University, Ganzhou, Jiangxi, China; ^3^ Department of Otorhinolaryngology, The Affiliated Fengcheng Hospital of Yichun University, Fengcheng, Jiangxi, China

**Keywords:** microbial metabolites, tumor immunity, immunometabolism, tumor microenvironment, immunotherapy

## Abstract

While tumor immunotherapy has achieved remarkable progress in many hematological malignancies, its efficacy remains limited by key challenges, including the immunosuppressive microenvironment of solid tumors, metabolic abnormalities, and drug resistance. As a central mechanism underlying impaired immune function, metabolic reprogramming of immune cells has emerged as a pivotal focus for unraveling tumor immune evasion and therapeutic resistance. Advances in metagenomics have highlighted the significance of the human commensal microbiome as a ‘second genome.’ Microbial metabolites, whether circulating systemically or accumulating locally, serve as key messengers linking the microbiota to tumor immunometabolism. This review comprehensively examines the regulatory roles and metabolic mechanisms through which microbial metabolites—including short-chain fatty acids (SCFAs), bile acids, tryptophan metabolites, and lipopolysaccharides (LPS)—modulate tumor immunity and immunotherapeutic responses via immune cell metabolism. These metabolites shape the tumor immune microenvironment and influence immunotherapeutic efficacy by reprogramming immune cell metabolic and biosynthetic pathways. This review underscores the central regulatory role of microbial metabolites as the ‘second genome’ in tumor immunometabolism, offering a theoretical foundation and potential targets to elucidate mechanisms of immunotherapeutic resistance and advance microbiota metabolism-based precision interventions.

## 1 Introduction

From the late 19th century to the mid-20th century, William Coley (1), Lloyd J. Old (2), and other researchers demonstrated that bacterial-induced immune activation, including *Bacillus Calmette-Guérin*, could promote tumor regression, thereby establishing the foundation of tumor immunotherapy. Subsequent breakthroughs, including cytokines (3, 4), monoclonal antibody technology (5, 6), immune checkpoint inhibitors (7), and bispecific antibodies, have significantly advanced tumor immunotherapy, particularly for hematological malignancies. For example, recent clinical trials (8, 9) have shown that CAR-T cell therapy with lisocabtagene maraleucel yields a 97% objective response rate in follicular lymphoma, with 81.9% of patients maintaining remission at 12 months and demonstrating reduced toxicity relative to conventional immunotherapies. Nonetheless, tumor immunotherapy still faces multiple challenges. In solid tumors, the dense extracellular matrix and immunosuppressive cell populations create both physical and cellular barriers within the tumor microenvironment (TME) (10). Within the TME, immune cells interact via complex signaling networks that collectively establish an immunosuppressive niche. Macrophages are key regulators within the TME and exhibit significant functional plasticity (11). Classically activated M1 macrophages exert anti-tumor effects through the secretion of pro-inflammatory cytokines, including TNF-α and IFN-γ. Conversely, alternatively activated M2-type tumor-associated macrophages (TAMs) suppress immune responses by producing factors such as IL-10 and TGF-β, and they promote tumor angiogenesis and metastasis. The binding of surface PD-L1 to PD-1 on T cells is a key mechanism driving T cell exhaustion (12). As professional antigen-presenting cells, dendritic cells (DCs) play a crucial role in activating naive T cells. Surface expression of MHC-peptide complexes and costimulatory molecules (e.g., CD80/86) provides the core signals required for T cell receptor (TCR) activation and the initiation of adaptive immune responses (13). The functional state of T cells directly determines the efficacy of anti-tumor immunity; however, their effector functions are subjected to multiple inhibitory mechanisms within the TME. This dysfunction arises not only from TAM-expressed PD-L1 but also from impaired infiltration due to abnormal angiogenesis and the presence of immunosuppressive cells such as myeloid-derived suppressor cells (MDSCs), which collectively inhibit T cell function (14). B cells also contribute to immune responses within tertiary lymphoid structures (TLSs). These ectopically formed lymphoid tissues contain T cell zones, B cell follicles, and germinal centers, within which B cells can regulate local immunity via antigen presentation, antibody production, and cytokine secretion (15). MDSCs deplete local essential amino acids and produce immunosuppressive metabolites via enzymes such as arginase 1 (ARG1) and inducible nitric oxide synthase (iNOS), thereby potently inhibiting CD8^+^ T cell proliferation and cytotoxic function. As such, MDSCs are considered core mediators of immune suppression within the TME (14, 15). Collectively, these cells and their signaling pathways form a complex regulatory network that governs immune responses within the TME. However, the loss or mutation of tumor-associated antigens and aberrant signaling pathway activation contribute to tumor antigen heterogeneity and drug resistance (16, 17). Furthermore, persistent microenvironmental stressors—such as hypoxia, elevated lactate levels, reactive oxygen species (ROS), and glucose deprivation—reprogram T-cell metabolism, impairing mitochondrial function and disrupting critical pathways, including oxidative phosphorylation (OXPHOS) and glycolysis (18, 19). These metabolic dysfunctions represent a key mechanism underlying T-cell exhaustion, diminished proliferation, and impaired effector molecule secretion, ultimately contributing to immunotherapy resistance. Thus, investigating immune cell metabolism in the tumor microenvironment may elucidate the fundamental mechanisms driving tumor immune evasion and immunotherapeutic resistance, offering potential therapeutic targets and a scientific foundation for refining immunotherapy strategies.

The advent of high-throughput sequencing technologies in the early 21st century enabled comprehensive metagenomic analyses, including 16S rRNA gene sequencing and whole-genome sequencing, to characterize the human commensal microbiome. These developments initiated the “second genome” concept and established microbiome research as a distinct scientific discipline (20). The microbiome comprises the collective microbial communities in a given environment, their genetic material, metabolic products, and dynamic interactions with the host ecosystem (21). This field has rapidly evolved into a central research paradigm in life sciences, comparable in significance to the Human Genome Project. The gut microbiota, representing the most substantial microbial community within the human microbiome, comprises diverse microorganisms - including bacteria and fungi - that colonize the intestinal tract (22). Contemporary research has demonstrated that the gut microbiota plays dual roles in both regulating host metabolic processes and shaping immune system development through microbial metabolites such as short-chain fatty acids (SCFAs) and lipopolysaccharides (LPS) (23). Furthermore, extensive evidence has established causal relationships between specific gut microbial taxa and various aspects of human health and disease pathogenesis (24). Notably, gut microbiota dysbiosis not only serves as a biomarker for disease initiation and progression (particularly in oncology) but also actively influences clinical outcomes, as demonstrated by specific microbial signatures like *Akkermansia muciniphila* and *Parabacteroides distasonis* in disease prognosis (25, 26). These findings collectively position the gut microbiota as a high-priority research focus with significant therapeutic potential. In addition to the gut microbiota, the tumor-associated microbiome has emerged as a critical area of investigation in oncology. Notably, microorganisms have been identified within diverse tumor types, including historically ‘sterile’ malignancies such as prostate and bladder cancers (27). These microbial communities influence oncogenesis and tumor progression through three primary mechanisms: (1) genotoxic effects promoting somatic mutations (27); (2) activation of oncogenic pathways, as demonstrated by riboflavin-mediated ceramide synthase inhibition (28); and (3) immunomodulation of the tumor microenvironment (29). Beyond direct cellular interactions, microbial metabolites—bioactive byproducts of microbial metabolism—serve as critical signaling mediators within the tumor microenvironment. These metabolites establish a tripartite signaling axis connecting host physiology, microbial communities, and malignant cells, fundamentally shaping tumor development and therapeutic responses (30, 31). This review systematically integrates contemporary evidence regarding microbiota-derived metabolites and their implications for tumor immunity and immunotherapy, with particular emphasis on their mechanistic basis: specifically, the metabolic reprogramming of immune cells through microbial metabolite signaling, which collectively determine antitumor immune responses and clinical immunotherapy efficacy. Through its focus on immunometabolic regulation, this work provides a comprehensive framework for understanding microbial metabolite-mediated control of antitumor immunity, thereby identifying novel therapeutic avenues for clinical translation in cancer immunotherapy.

## 2 The central role of metabolism in shaping immune cell fate and function

The efficacy of immune responses fundamentally depends on both the abundance and functional competence of immune cells. Compromised immune cell function significantly impairs immune surveillance, promoting tumor immune evasion and contributing to immunotherapy resistance. Immunometabolism research examines the bidirectional relationship between metabolic pathways (including OXPHOS and glycolysis) and immune cell function, while also investigating how immune cells regulate tissue-specific metabolic homeostasis (32). Beyond energy production, cellular metabolism generates essential signaling molecules and biosynthetic precursors that orchestrate immune cell function (33). A comprehensive understanding of immunometabolic regulation requires elucidation of both cell-intrinsic metabolic programs and their functional consequences for immune cell differentiation and effector responses.

### 2.1 Metabolic substrates: the energy and biosynthetic foundation of immune cell function

Immune cells, like all human cells, require continuous energy metabolism and biosynthetic precursors to sustain their viability and functional capacity. This demand is met through the integrated operation of complex metabolic networks. ATP serves as the fundamental energy currency that drives essential immune cell activities including migration, phagocytosis, and cytokine secretion. For instance, T cell migration to inflammatory sites following activation depends on ATP-dependent cytoskeletal remodeling, where actin-myosin interactions facilitate cellular movement (18, 34). Similarly, macrophage phagocytic function - encompassing phagosome formation, proton pump-mediated acidification, and antimicrobial enzyme activation - is fundamentally energy-dependent (35). These examples underscore how metabolic activity represents a critical determinant of immune cell functional capacity, with ATP availability serving as a key rate-limiting factor in immune effector responses.

The availability of biosynthetic precursors is equally vital for supporting immune cell proliferation and effector functions. During immune activation, the clonal expansion of lymphocytes requires rapid biosynthesis of macromolecules including nucleic acids, proteins, and membrane constituents. This process critically depends on several interconnected metabolic pathways: *De novo* nucleotide synthesis utilizes glycine and aspartate as nitrogen donors while incorporating ribose-5-phosphate from the pentose phosphate pathway to form purine skeletons (36). Amino acid metabolism plays particularly versatile roles, with glutamine serving dual functions - fueling the TCA cycle for energy production while simultaneously providing nitrogen for nucleotide and amino acid biosynthesis (37, 38). In macrophages, arginine metabolism generates nitric oxide, a potent antimicrobial and antitumor effector molecule (39). Lipid metabolism serves essential structural and functional roles in immune cell biology. Phospholipid biosynthesis is markedly upregulated in activated immune cells to support membrane biogenesis during proliferation (40, 41). Beyond membrane formation, lipid metabolism contributes to immune cell function through multiple mechanisms: fatty acid β-oxidation generates ATP to sustain memory T cell persistence, while cholesterol-derived metabolites regulate critical signaling pathways (42). These metabolic pathways form an intricately interconnected network that maintains dynamic equilibrium in immune cells. Disruption of this metabolic homeostasis - whether through substrate limitation or pathway dysregulation - leads to profound immune dysfunction. For example, T cells experiencing metabolic stress demonstrate impaired migratory capacity due to energy deficits, while biosynthetic precursor shortages suppress proliferative responses. Such metabolic perturbations can tip the immune balance toward either immunodeficiency or pathological inflammation, representing key mechanistic drivers in diverse disease states including infections, autoimmune disorders, and malignancies (18).

### 2.2 Immune cell metabolic reprogramming: core hub for regulating immune responses and cell fate

The plasticity of immune cell metabolism is a fundamental mechanism that not only enables immune cells to adapt to different functional states but also allows them to actively regulate immune responses. Metabolic reprogramming supplies energy and biosynthetic precursors for immune cells and directly influences cell differentiation and function by regulating signal transduction, epigenetic modifications, and gene expression. In T cells, upon activation, effector T cells (Teff) rapidly upregulate glycolysis to meet the demands of proliferation and the synthesis of effector molecules. During this process, the costimulatory CD28 signal promotes the expression of the pyruvate kinase M2 (PKM2) isoform via the Ars2 protein, thereby enhancing glycolytic flux. Furthermore, the glycolytic enzyme GAPDH functions as an RNA-binding protein: when glycolysis is active, it binds to the 3’UTR of IFN-γ mRNA to inhibit its translation, but dissociates under glucose restriction to promote IFN-γ production, thereby constituting a dynamic “metabolism-translation” switch (43). Additionally, SREBPs activate cellular lipid synthesis pathways, providing essential lipids for membrane expansion and for signal transduction in Teff cells; defects in this process lead to impaired clonal expansion and functional loss (44). Organelle interactions also play a key role in metabolic regulation. Mitochondria balance the metabolic needs of Teff and memory T cells through fission and fusion: Drp1-mediated mitochondrial fission promotes glycolytic metabolism in Teff, while mitochondrial fusion, maintained by Opa1, supports OXPHOS in memory T cells (45). Furthermore, lysosomes maintain metabolic homeostasis by clearing damaged mitochondria via autophagy. Additionally, the breakdown of fatty acids, driven by lysosomal acid lipase (LAL), provides fatty acids and cholesterol for memory T cells, supporting their long-term survival (45). Metabolic programs further regulate T cell fate through epigenetic mechanisms. Acetyl-CoA, a key substrate for histone acetylation, whose metabolic source determines the functional state of T cells. Teff cells primarily rely on ACSS2 to generate acetyl-CoA from acetate, thereby maintaining high levels of histone acetylation that promote the expression of effector genes such as IFN-γ and TNF-α. However, in exhausted T cells (Tex), ACSS2 expression is significantly downregulated, and these cells instead rely on ACLY to generate acetyl-CoA from citrate; however, this metabolic pathway sustains only the expression of exhaustion-related genes and cannot support full effector function (46). This metabolic-epigenetic coupling is particularly prominent in the tumor microenvironment. For example, methionine deficiency inhibits arginine methylation of the potassium channel KCa3.1, leading to abnormal calcium ion influx and excessive activation of NFAT1 signaling, which directly drives T cell differentiation toward an exhausted phenotype (46).

Under normal physiological conditions, distinct metabolic characteristics define the functional properties of various immune cell subsets. For instance, regulatory T cells (Tregs) primarily rely on OXPHOS and fatty acid oxidation (FAO) to maintain their suppressive function (47, 48). In the tumor microenvironment, Treg cells efficiently take up lactate via the monocarboxylate transporter MCT1, convert it to pyruvate through lactate dehydrogenase for entry into the TCA cycle, and leverage FAO to maintain survival and function under nutrient-poor conditions (47). HIF-1α plays a key regulatory role in this process: Stabilized by glycolytic metabolism, HIF-1α binds to the CD73 promoter to induce its high expression, thereby enabling Treg cells to convert extracellular AMP into immunosuppressive adenosine and significantly enhance their immunosuppressive capacity (49). In contrast, Th17 cell differentiation depends on HIF-1α-driven glycolysis and glutaminolysis, which promote IL-17 secretion through the activation of the transcription factor RORγt (50). Memory T cells mount rapid responses through a unique mechanism: upon TCR activation, downstream signaling molecules ZAP70 and LCK directly phosphorylate glycogen phosphorylase B (PYGB), which catalyzes the breakdown of glycogen into glucose-1-phosphate. This facilitates the rapid initiation of glycolysis even in the absence of exogenous glucose, thereby supporting a rapid recall response (51). A portion of the glucose-6-phosphate produced by glycogen breakdown enters the pentose phosphate pathway, generating NADPH to maintain redox homeostasis and ensure proper effector molecule synthesis (51). Under pathological conditions, immune dysfunction is closely linked to cellular metabolic abnormalities. T cell exhaustion, induced by chronic infection and the tumor microenvironment, manifests as a vicious cycle in which metabolic defects and epigenetic changes mutually reinforce each other. This state is characterized by impaired mitochondrial function, low glycolytic efficiency, and a global decrease in histone acetylation levels. Notably, the reduction of H3K27ac is closely associated with the silencing of exhaustion-related genes (46, 52).

## 3 Regulation of microbial metabolites on tumor immunity and immunotherapy

A growing body of clinical and experimental evidence has underscored the pivotal role of the microbiota in tumor initiation, progression, and therapeutic responses. First, significant differences in the composition and functional profiles of microbial communities have been identified between cancer patients and healthy individuals across multiple niches, including the gut, tumor tissues, blood, and adjacent normal tissues (53–55). These differences are not limited to local microbiota but also exert systemic effects on immune regulation and distant microbial communities via the circulatory system (56, 57), thereby establishing a complex tumor-microbiota-immune network. Second, deeper mechanistic investigations have revealed that active modulation of the microbiota—particularly the gut microbiota—through interventions such as fecal microbiota transplantation (FMT), probiotics, or antibiotics can significantly influence tumor progression and enhance clinical outcomes (58). Most notably, both the composition and diversity of the microbiota profoundly impact therapeutic efficacy, particularly in the context of immunotherapy (59). The regulatory impact of microbiota on tumor initiation, progression, and treatment responses is predominantly mediated through precise modulation of host immune system (53). First, microbiota influences antigen presentation efficiency and regulates the balanced secretion of inflammatory cytokines by controlling myeloid cell differentiation and function (49). Furthermore, microbiota shapes the tumor immune microenvironment by orchestrating the activation, proliferation, and tissue infiltration of T cell subsets (60, 61). However, a critical question arises when investigating how the microbiota regulates immune cell metabolism: beyond direct microbial colonization of tumors, how do most microorganisms—particularly gut microbiota—influence immune cell metabolism in systemic or distant tumor microenvironments? Evidently, the microbiota’s direct effects alone cannot fully account for this phenomenon. Laura et al. (62)demonstrated that *in vitro* supplementation with *Akkermansia*, as measured by plasma metabolite concentrations, neither translocates to other organs nor displaces other microbial populations. Instead, *Akkermansia* metabolizes tryptophan into indoles within the gastrointestinal tract; these metabolites then enter systemic circulation and reach the tumor microenvironment, where they modulate CAR-T cell recognition and cytotoxic activity against tumors. Furthermore, two independent untargeted liquid chromatography-based metabolomics studies identified significant differences in host metabolites between microbiota-intervened groups and immunotherapy responders/non-responders (63, 64). Thus, microbiota-derived metabolites likely serve as the primary mediators regulating tumor immunometabolism. These metabolites can cross the intestinal mucosal barrier, entering systemic circulation or accumulating locally within tumors, where they influence immune cells via receptor-dependent or -independent mechanisms to modulate key signaling pathways. Below, we summarize several major metabolites that impact immune cell function in the tumor microenvironment.

### 3.1 Short-chain fatty acids

SCFAs primarily consisting of acetate, propionate, and butyrate, represent a crucial class of metabolites generated by gut microbiota through the catabolism of complex carbohydrates such as dietary fiber (65). Extensive evidence has demonstrated their pleiotropic functions in maintaining human health: they act not only as a major energy substrate for specific cell types (e.g., intestinal epithelial cells) (66), but also exhibit significant immunomodulatory properties with implications for tumor immunity (67). Liu et al. (68) demonstrated that gut microbial metabolites—particularly butyrate—and their synthetic analogs significantly impact long-term therapeutic outcomes and adverse event profiles in lung cancer survivors by modulating both immune responses and the tumor microenvironment, thereby influencing lung cancer initiation, progression, and post-treatment prognosis. A clinical study (69) compared the gut microbiota of melanoma patients who responded to anti-PD-1 therapy with that of non-responders. The analysis revealed that responders exhibited greater microbial richness and a higher abundance of SCFA-producing Clostridiale*s* bacteria. Subsequent analyses demonstrated that these microbial communities enhance immunotherapy efficacy by increasing SCFA levels. Further studies evaluating responses to FMT revealed that FMT responders had an increased intestinal abundance of SCFA-producing bacteria, a feature directly associated with enhanced efficacy of anti-PD-1 therapy (70). These findings underscore the important role of SCFAs in modulating antitumor immunity. In complementary research, scientists isolating SCFAs produced by *Escherichia coli* from healthy human gut microbiota observed that these metabolites may inhibit cancer progression through metabolic reprogramming of tumor cells, suppression of pro-inflammatory signaling pathways, and regulation of immune cell functions (71). Further investigations (72) have established that gut microbiota-derived SCFAs foster a tumor-permissive microenvironment by inducing cancer cell autophagy and promoting M2 macrophage polarization. Notably, butyrate has been specifically shown to accelerate tumor progression by modifying cancer cell behaviors—including enhanced migratory and invasive capacities—while simultaneously reshaping the tumor microenvironment to support angiogenesis and immune evasion mechanisms (73). Concurrently, studies have investigated the direct effects of SCFAs on immune cells. For example, Camille Danne et al. (74, 75) examined the therapeutic potential of butyrate in cancer treatment by analyzing its modulation of CD8^+^ T cell immune responses and its ability to downregulate immunosuppressive factors (PD-L1 and IL-10) in TAMs. Furthermore, SCFAs have been shown to regulate the proliferation, differentiation, and activity of type 3 innate lymphoid cells, consequently shaping the hepatic immune microenvironment and significantly influencing hepatocarcinogenesis, tumor progression, and immune evasion mechanisms (76).

Numerous studies (77, 78) have demonstrated that modulation of microbiota-derived SCFA levels through various approaches can significantly influence tumor immunity by regulating the host immune system (**Table 1**). Generally, pharmaceutical or dietary interventions that elevate SCFA levels—including acetate, propionate, butyrate, and valerate—affect the activity of immune cells such as macrophages, T cells, and NK cells, ultimately altering the tumor immune microenvironment. These alterations are primarily manifested through changes in immune cell infiltration patterns and cytokine profiles, which subsequently impact clinical treatment outcomes. For instance, butyrate and SCFA-A have been shown to potentiate the effectiveness of anti-PD-1 therapy in cancer patients by activating tumor-infiltrating GNLY^+^ CD8^+^ T cells and promoting T cell activation, respectively, as well as by inducing M1 macrophage polarization (79, 80). Sodium butyrate has been demonstrated to convert immunologically “cold” tumors into “hot” tumors through regulation of gut microbiota-derived SCFAs, thereby enhancing immunotherapy response (81). Moreover, Yang et al. revealed that gut microbiota-derived butyrate compromises the anti-tumor efficacy of ionizing radiation by suppressing TBK1 and IRF3 phosphorylation in DCs, inhibiting STING-dependent type I interferon production, and consequently impairing tumor-specific cytotoxic T cell responses (82). Collectively, gut microbiota-derived SCFAs exhibit context-dependent dual roles in cancer progression through their immunomodulatory effects on both immune cell function and tumor microenvironment dynamics, ultimately determining anti-tumor immune responses and clinical treatment outcomes.

**Table 1:** Overview of short-chain fatty acids on immune cells.

Intervention	SCFAs	SCFAs levels	Target cells	Functions	Reference
Adaptogenic flower buds	Butyrate	increase	Macrophage	Promote macrophages to M2 type Promote inflammatory factors release	(77)
Icariside I	Acetate Propionate Butyrate Valerate	increase	CD4^+^ T cells CD8^+^ T cells NK cells NKT cells	Increase level of CD4^+^ T, NK and NKT cells Promote release of IL-6 and IL-1β	(83)
Ganoderma lucidum polysaccharide	not mentioned	increase	Macrophage	Decrease level of macrophage Inhibit release of IL-1β, iNOS and COX-2.	(84)
Inulin gel	Acetate Propionate Butyrate	increase	CD8^+^ T cells	Enhanced CD8^+^ T cells response Enhances PD-1 therapeutic effect	(85)
Naturalizing	Acetate Butyrate	increase	CD4^+^ T cells CD8^+^ T cells NK cells Tregs	Promote immune maturation of T and NK cells	(86)
Traditional Patchouli essential oil	not mentioned	increase	Macrophage CD4^+^ T cells CD8^+^ T cells	Promote macrophages to M2 type Increase level of CD4^+^ T and CD8^+^ T cells	(87)
Polysaccharides from Tetrastigma hemsleyanum	Acetate Propionate Butyrate Valerate	increase	CD4^+^ T cells CD8^+^ T cells NK cells	Enhanced NK cells response Increase level of CD4^+^ T cells	(88)
Holothuria leucospilota Polysaccharides	Acetate Propionate	increase	not mentioned	Promote release of IL-2, IL-6, IL-17, IFN-γ, TNF-α, IgG, IgM, and IgA	(89)
Caloric restriction	Acetate	increase	CD8^+^ T cells	Increase level of CD8^+^ T cells Promote release of IFN-γ	(90)
Curcumin	not mentioned	increase	CD8^+^ T cells	Increase level of CD8^+^ T cells	(91)
Mannose	Propionate Butyrate	increase	CD8^+^ T cells	Increase level of CD8^+^ T cells	(92)
Sodium Butyrate	not mentioned	increase	CD4^+^ T cells CD8^+^ T cells	Increase level of CD4^+^ T cells Promote release of IFN-γ	(81)
High-fiber diets	not mentioned	increase	Tregs	Promote inflammatory factors release Increase level of CD4^+^Foxp3^+^RORγt^+^IL-17A^+^ regulatory T cells	(78)

SCFAs, short-chain fatty acids; iNOS, inducible nitric oxide synthase; COX-2, cyclooxygenase-2; PD-1, programmed death receptor 1; IFN-γ, interferon-γ; IL, interleukins; TNF-α, tumor necrosis factor-α.

### 3.2 Bile acids

Bile acids, a class of microbiota-modified metabolites derived from hepatic cholesterol metabolism, serve as crucial signaling molecules that regulate host physiological processes including lipid metabolism, glucose homeostasis, and immune responses (93). Emerging evidence underscores their significant contributions to tumor immunomodulation and immunotherapy efficacy. Shaikh et al. (94) examined the relationship between fecal microbiome composition and pathological complete response in resectable esophageal cancer patients undergoing combined chemoradiotherapy and immunotherapy. Through 16S rRNA amplicon sequencing of fecal samples, they identified distinct microbial signatures in responders, which were associated with metabolic pathways including secondary bile acid biosynthesis. These findings suggest that microbiome-derived bile acids may represent potential biomarkers for treatment response prediction. In a complementary study (95), researchers demonstrated that gut microbiota-generated bile acids activate TGR5 signaling, thereby promoting colorectal cancer liver metastasis via recruitment of MDSCs to the hepatic microenvironment. This work elucidates the gut microbiota-bile acid-TGR5-MDSC axis as a critical mediator of colorectal cancer metastasis. From a molecular mechanistic standpoint, gut microbiota-mediated metabolism of primary to secondary bile acids can suppress CD8^+^ T cell effector functions, thereby promoting cancer cell proliferation and invasion (96). Conversely, bile acid signaling through intestinal epithelial cell receptors modulates MAdCAM-1 expression, which regulates gut-specific lymphocyte homing and consequently influences intestinal immune surveillance and cancer development (97). Notably, pharmacological agents including traditional compounds (*Xianglian pill* and *Huaier polysaccharides*) have been shown to remodel gut microbial communities and bile acid metabolic profiles, resulting in suppressed tumor growth and diminished immune evasion through reduction of M2 macrophage polarization (98, 99). Notably, Ji et al. (100) employed nanotechnology for targeted hepatic delivery of bile acid receptor modulators, achieving precise regulation of bile acid signaling pathways. This intervention induced immune cell activation, significantly enhancing tumor and hepatic infiltration of NKT, NK, CD4^+^, and CD8^+^ T cells while elevating cytokine and chemokine levels in both serum and tumor microenvironments - establishing a novel immunotherapeutic approach for hepatocellular carcinoma. Collectively, these findings position gut microbiota-derived bile acids as pivotal immunomodulators in both cancer pathogenesis and treatment response. Their dual functionality in facilitating metastatic progression while simultaneously potentiating immunotherapy efficacy highlights the microbiota-bile acid axis as both a promising biomarker and therapeutic target in oncology.

### 3.3 Tryptophan metabolites

As an essential amino acid in humans, tryptophan is not only utilized by the host for protein synthesis and metabolized via its intrinsic kynurenine pathway but also undergoes substantial breakdown and transformation by the gut microbiota through a unique set of enzymatic systems, generating a diverse array of metabolites with distinct structures and functions (101). Acting as chemical messengers, these metabolites profoundly regulate host intestinal homeostasis, immune balance, neurological function, and systemic metabolic health (102, 103). These metabolites are primarily synthesized through three core metabolic pathways: the ubiquitous indole pathway, in which tryptophanase cleaves tryptophan to generate indole and its derivatives (e.g., indole-3-propionate); the tryptophan-tryptamine pathway, producing tryptamine via decarboxylation; and the microbial kynurenine pathway, yielding metabolites such as kynurenine and kynurenic acid (104). Mirroring the roles of other microbial metabolites, tryptophan-derived compounds significantly modulate host antitumor immune responses.

**Figure 1:**
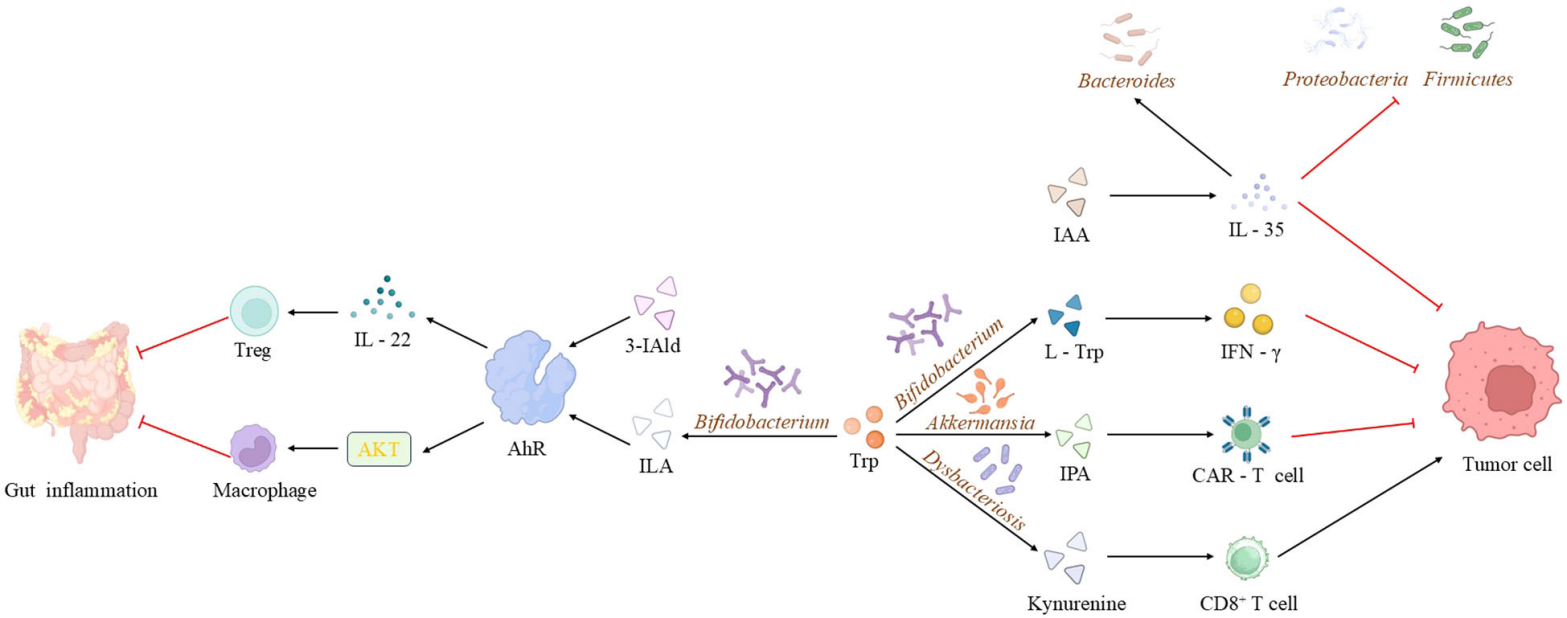
Mechanisms of microbiota-derived tryptophan metabolites in disease pathogenesis. Microbiota-derived tryptophan metabolites (e.g., IAA, IPA, 3-IAld, indole-3-lactic acid) contribute to disease processes through multiple pathways: IAA ameliorates precancerous intestinal inflammation and modulates the microbiota via IL-35; metabolites derived from *Bifidobacterium* enhance the synthesis of immune molecules, synergizing with anti-tumor therapies; supplementation with *Akkermansia* spp. and IPA influences CAR-T cell efficacy; 3-IAld and *Bacteroides* spp. potentiate immunotherapy and suppress inflammation via the AhR/IL-22 axis and Treg cell induction; while kynurenine, produced during oral dysbiosis, impairs CD8^+^ T cell function and promotes tumor progression. (IAA, indole-3-acetic acid; IPA, indole-3-propionic acid; 3-IAld, 3-indolealdehyde; ILA, Indole-3-lactic acid).

Accumulating evidence (105, 106) indicates that gut microbiota-derived tryptophan metabolites, including indole-3-acetic acid and indole-3-lactic acid (ILA), suppress tumor initiation and progression through modulation of the precancerous inflammatory microenvironment and regulation of tumor cell energy metabolism. These metabolites also contribute significantly to tumor immunoregulation. In a clinical study, Lee et al. (107) observed enrichment of *Bifidobacterium* in the gut microbiota of immunotherapy-responsive non-small cell lung cancer patients. Complementary murine studies demonstrated that specific *Bifidobacterium* strains potentiated the efficacy of both anti-PD-1 therapy and oxaliplatin, significantly reducing tumor burden through enhanced antitumor immune responses. Mechanistic investigations revealed that *Bifidobacterium*-mediated upregulation of immunostimulatory molecules and L-tryptophan biosynthesis promotes interferon-γ production, thereby modifying the immune microenvironment to achieve these therapeutic effects. Another study (62) showed that modulating the gut microbiota via supplementation with *Akkermansia* spp increased CAR-T cell efficacy, with plasma concentrations of indole metabolites in the tryptophan pathway exhibiting statistically significant temporal changes. These findings collectively suggest that tryptophan metabolites act as microbial “messengers” with potential tumor immunoregulatory roles. In studies of cancer immunotherapy, multiple investigations (108) have indicated that gut microbiota-derived tryptophan metabolites may enhance the efficacy of immune checkpoint inhibitors by regulating the function of immune cells and immune signaling pathways within the tumor microenvironment. Emerging evidence (109–111) indicates that dietary modifications—including high-salt diets, ginseng polysaccharide supplementation, or fucoidan administration—can reshape gut microbial communities and optimize tryptophan metabolism. This microbial remodeling subsequently adjusts tryptophan metabolite levels, thereby modulating immune-related metabolic pathways to potentiate antitumor immunity. Importantly, extraintestinal microbiota-derived tryptophan metabolites exhibit comparable immunomodulatory effects. Chen et al. (112) reported that in advanced non-small cell lung cancer, dysbiosis of the lower respiratory tract microbiota disrupts local tryptophan metabolism, consequently altering the pulmonary immune microenvironment, immune cell functionality, and tumor immunogenicity—factors that collectively determine anti-PD-1 treatment response. Parallel findings demonstrate that stress-induced oral microbiota dysbiosis elevates kynurenine production, which remodels the tumor immune landscape by suppressing CD8^+^ T cell activity while promoting proliferation and metastasis in head and neck squamous cell carcinoma (113). In contrast to conventional microbial formulations that face challenges including low viability and inadequate colonization/targeting efficiency, nanoengineered microbial preparations have demonstrated substantial therapeutic advances. Nanotechnology-enabled microbial delivery systems facilitate precise intestinal colonization of selected gut microorganisms, which subsequently modulate tryptophan metabolism to generate immunologically active indole derivatives. These microbial metabolites directly influence tumor-infiltrating immune cells, reprogramming their functional states to potentiate antitumor responses and suppress both primary tumor growth and metastatic dissemination (114, 115). Importantly, the immunomodulatory effects of microbiota-derived tryptophan metabolites are context-dependent - while many enhance immune function, certain metabolites like indolepropionic acid can exert immunosuppressive activity (**Figure 1**). This functional duality positions tryptophan metabolites as pleiotropic immune modulators that fine-tune immune cell behavior and response magnitude to maintain systemic immunologic homeostasis (116). Results from a Phase III clinical trial demonstrated that, compared to a placebo control, combining the IDO1 inhibitor epacadostat with the PD-1 inhibitor pembrolizumab did not significantly improve progression-free survival or overall survival in melanoma patients. These results indicate that solely inhibiting the IDO1 enzyme is likely insufficient, highlighting the need for a more comprehensive understanding of the complexities of the broader tryptophan metabolic network (117).

### 3.4 Lipopolysaccharides

LPS, essential structural components of Gram-negative bacterial outer membranes, are released into the host environment during microbial growth, division, and lysis. As highly bioactive molecules, LPS serves as a potent signaling mediator that significantly modulates both physiological and pathological host states. Given its critical role in microbe-host crosstalk, LPS is frequently classified among biologically significant “microbiota-derived metabolites” or “microbe-associated molecular patterns” (118). The initial evidence connecting microbial LPS to tumorigenesis stems from its role in chronic inflammation regulation. Wu et al. (119) demonstrated that *Bacteroidetes*-derived LPS activates the TLR4/MyD88/NF-κB signaling cascade, inducing chronic inflammatory responses that facilitate esophageal squamous cell carcinoma invasion and metastasis. Subsequent research has increasingly highlighted LPS’s immunomodulatory potential. In oncological contexts, research has revealed significant associations between LPS-related biomarkers and tumor progression. A clinical study (120) demonstrated that LPS-binding protein expression levels correlate with metastatic progression in osteosarcoma patients, potentially influencing tumor cell invasiveness through modulation of inflammatory responses and immune cell activity. Complementary work (121) established that gastric cancer development and progression are strongly linked to bacterial LPS-associated genes, identifying these genetic markers as potential prognostic indicators and predictors of immunotherapy response. Furthermore, preclinical investigations using a murine melanoma model (122) showed that *Lactobacillus plantarum* WLPL09-derived exopolysaccharides suppress tumor growth and metastasis via immunomodulatory mechanisms that enhance immune cell effector functions. Within tumor immunotherapy, LPS - like other microbiota metabolites discussed - enhances host antitumor immunity by activating specific immune cell populations and intracellular signaling pathways, thereby potentiating immune checkpoint blockade efficacy including anti-PD-1 therapy (123–125). These collective findings establish LPS as a crucial microbe-associated molecular pattern that orchestrates multiple oncological processes, from tumor initiation and metastatic progression to therapeutic response modulation, primarily through its dual regulation of chronic inflammation and immune system function.

## 4 Metabolic reprogramming of tumor-associated immune cells by microbiota-derived metabolites: mechanisms and therapeutic implications

Drawing upon established knowledge of metabolic control in immune cell function and the immunomodulatory capacity of microbiota metabolites in tumor immunity, this review examines the specific metabolic pathways through which microbial metabolites influence immune cell antitumor activity. We focus on critical pathways including glycolysis, OXPHOS, the pentose phosphate pathway, fatty acid metabolism, and amino acid metabolism. Elucidating how microbiota metabolites reprogram these interconnected metabolic networks in immune cells will provide deeper mechanistic understanding of microbial immunomodulation while revealing novel therapeutic targets to address immunotherapy resistance. These insights may further guide the development of microbiota-based approaches to enhance metabolic fitness and improve clinical outcomes in cancer immunotherapy. Here, we systematically compare the mechanisms by which these metabolites regulate immune cell metabolism, focusing on their functional characteristics and potential therapeutic applications in anti-tumor immunity.

### 4.1 Short-chain fatty acids: synergistic regulation of immune cell metabolism and function via multiple mechanisms

SCFAs—primarily acetate, propionate, and butyrate—are produced by the anaerobic fermentation of dietary fiber by the intestinal microbiota. They exert their effects through multiple mechanisms: acting as histone deacetylase (HDAC) inhibitors to modulate gene expression epigenetically; serving as ligands for G protein-coupled receptors (GPCRs, GPR43, GPR41, and GPR109A) to initiate downstream signaling; and functioning as metabolic substrates that are directly integrated into cellular metabolic pathways to alter the metabolic state of immune cells. In regulatory lymphocytes, SCFAs promote the expression of anti-inflammatory factors by enhancing glycolysis. For example, pentanoate upregulates hexokinase 2 (HK2) expression in B cells to enhance glycolytic flux, thereby promoting IL-10 secretion (126). In Th17 cells, pentanoate utilizes a distinct mechanism—inhibition of AMPK and activation of the AKT/mTOR signaling axis—to enhance glycolysis and induce IL-10 production, highlighting the cell-type specificity of metabolic regulation. The effect of butyrate on CD4^+^ T cell subsets is more complex: it enhances glycolysis and mitochondrial oxidation by upregulating the glucose transporter Glut1, which prompts IL-10-producing Th1 cells to express granzyme B (GzmB) and thereby exerts a regulatory function in intestinal inflammation (127). Alternatively, the mechanism through which butyrate promotes Tregs differentiation is dependent on enhanced FAO. Specifically, butyrate is converted to butyryl-CoA by the ACSS2 enzyme; butyryl-CoA then alleviates the inhibition of carnitine palmitoyltransferase 1A (CPT1A) by antagonizing malonyl-CoA, thereby promoting FAO and providing metabolic support for iTreg differentiation (128).

**Figure 2:**
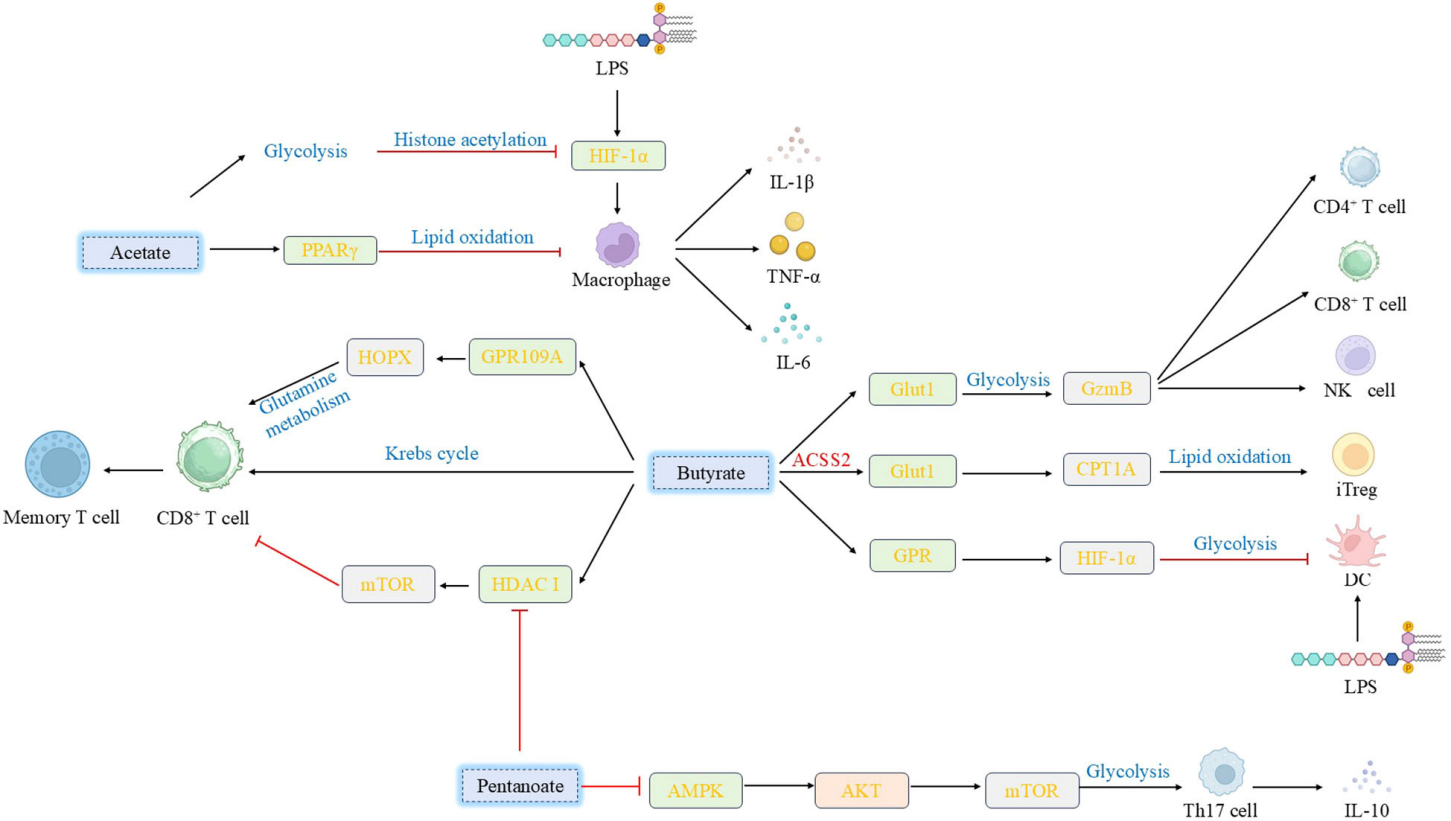
Effects of various short-chain fatty acids on immune cells. SCFAs exert pleiotropic regulatory effects on immune cells: Sodium acetate activates PPARγ to mitigate macrophage inflammation, whereas high-dose acetate suppresses macrophage glycolysis and immune responses by impacting HIF-1α activation and protein acetylation. Butyrate upregulates Glut1 to modulate Th1 cell metabolism, promoting GzmB expression; it also enhances CD8^+^ T cell memory potential and regulates iTreg cell differentiation via metabolic pathways. Furthermore, butyrate activates GPCRs or inhibits class I HDACs, thereby modulating dendritic cell inflammatory responses and regulating CD8^+^ T cell activation, proliferation, and antitumor efficacy. Valerate enhances IL-10 secretion by Breg cells and modulates Th17 cell function through regulation of glycolytic enzymes and the AKT/mTOR signaling pathway. (SCFAs, Short-chain fatty acids; PPARγ, peroxisome proliferator-activated receptor γ; GzmB, Granzyme B; iTreg, inducible T regulatory; GPCRs, G protein-coupled receptors; HDACs, histone deacetylases; IL-10, interleukin-10; Breg, regulatory B).

SCFAs also play critical roles in innate immune cells. Butyrate inhibits glycolysis in DCs by activating GPCRs and upregulating HIF-1α, thereby attenuating their inflammatory activation (129). In contrast, macrophage function is significantly influenced by SCFA concentration and exposure duration: high-dose acetate inhibits glycolysis and inflammatory responses by suppressing HIF-1α activity and promoting the acetylation of the p65 subunit, which reflects the dose dependency of metabolic regulation (130). Central to anti-tumor immunity, the key role of SCFAs stems from their potent ability to enhance the metabolism and function of CD8^+^ T cells. Through the inhibition of class I HDACs and sustained activation of mTOR signaling, butyrate and pentanoate significantly enhance the glycolytic capacity, proliferative potential, and secretion of effector molecules (e.g., IFN-γ and TNF-α) in CD8^+^ T cells, thereby improving the efficacy of adoptive T cell therapy (ACT) (131). Additionally, butyrate activates the HOPX signaling pathway through the GPR109 receptor, thereby participating in the regulation of glutamine metabolism and competition in CD8^+^ T cells, which further influences their immune function (132). Collectively, these studies demonstrate that SCFAs reprogram immune cell metabolism through multiple targets and mechanisms, thereby serving as powerful adjuvants in anti-tumor immunity (**Figure 2**).

### 4.2 Tryptophan metabolites: context-dependent immunometabolic regulation centered on the AhR signaling axis

Tryptophan metabolites constitute a critical interface between the microbiota and host immunity, and the balance or dysregulation of their metabolic pathways significantly influences anti-tumor immunity. Tryptophan is metabolized primarily through three pathways: the kynurenine pathway, the 5-hydroxytryptamine (5-HT) pathway, and the microbial indole derivative pathway (104). Among these, the kynurenine pathway is the most extensively studied in tumor immunology. Its key rate-limiting enzymes, indoleamine 2,3-dioxygenase (IDO) and tryptophan 2,3-dioxygenase (TDO), are highly expressed in various tumors, leading to tryptophan depletion and the accumulation of metabolites such as kynurenine in the tumor microenvironment (133). This “dual-hit” mechanism—comprising amino acid deprivation and toxic metabolite accumulation—collectively induces T cell cycle arrest and functional exhaustion, thereby fostering an immunosuppressive microenvironment. It is noteworthy that IDO, a pivotal enzyme in tryptophan metabolism and a critical immunoregulator, contributes not only to tumor immune suppression and progression but also to the pathogenesis of various non-tumor diseases. For example, IDO is highly expressed in the brains of Toxoplasma gondii-infected BALB/c mice, where it inhibits the proliferation of T. gondii tachyzoites by depleting tryptophan. In IDO-deficient hosts, this function can be compensated for by the upregulation of IFN-γ-mediated guanylate binding protein 1 (GBP1) and inducible nitric oxide synthase (NOS2), thereby maintaining anti-infective capacity (134). Furthermore, in autoimmune diseases, IDO exerts a protective effect by modulating excessive immune responses via multiple mechanisms: it restricts effector T cell activation, activates aryl hydrocarbon receptor (AhR) to promote Treg cell differentiation, and directly suppresses the release of inflammatory factors such as TNF-α and IL-6 (135, 136).

**Figure 3:**
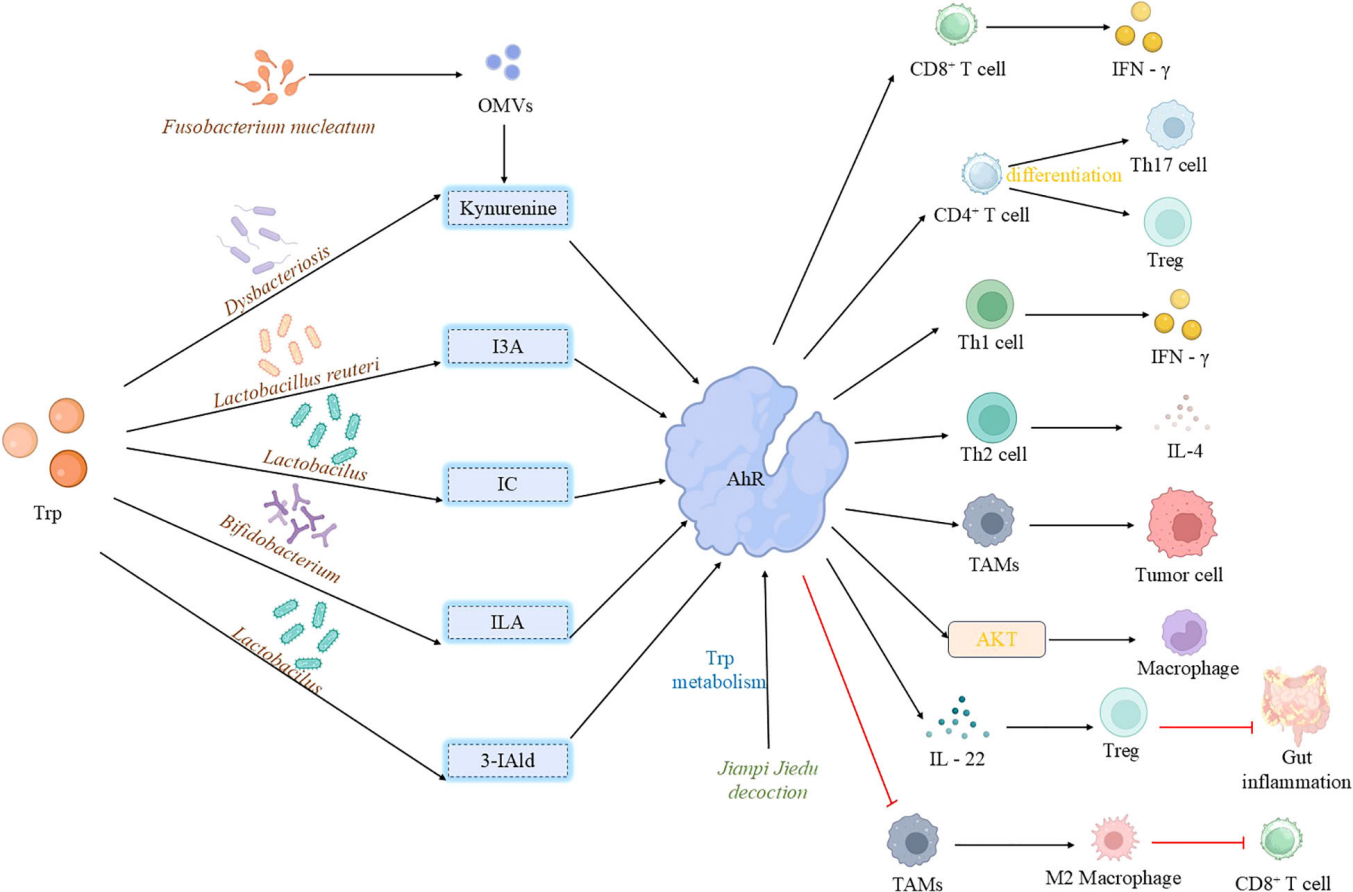
Activation of aryl hydrocarbon receptor by microbial tryptophan metabolites. Tryptophan-derived microbiota metabolites exhibit diverse effects on AhR activation: Indole, generated from tryptophan metabolism by *Lactobacillus*, activates AhR in TAMs, correlating with disease progression and immunosuppression. Furthermore, outer membrane vesicles from *Fusobacterium nucleatum* remodel the tryptophan metabolism pathway in TAMs, inducing an immunosuppressive microenvironment that drives resistance to tumor immunotherapy. Similarly, indole derivatives (ILA and IAld) produced by gut microbiota (primarily *Lactobacillus*) activate AhR in TAMs, promoting an immunosuppressive phenotype that suppresses CD8^+^ T cell anti-tumor activity and ultimately fosters the growth of pancreatic ductal adenocarcinoma.3-IAld strengthens the intestinal barrier and controls inflammation via the AhR/IL-22 axis. I3A from *Limosilactobacillus* reuteri activates AhR in CD8^+^ T cells, promoting IFN-γ production to enhance immunotherapy efficacy; it also modulates T cell differentiation through AhR. ILA, produced by *Bifidobacterium breve*, acts as an AhR ligand to protect the intestinal barrier, suppress inflammation, and regulate macrophage polarization. *Jianpi Jiedu Decoction* inhibits TAM polarization via the tryptophan metabolism-AhR pathway, while kynurenine, produced by oral microbiota under stress, activates AhR leading to CD8^+^ T cell exhaustion and promotion of tumor progression. (TAMs, tumor-associated macrophages; 3-IAld, 3-Indolealdehyde, I3A, indole-3-aldehyde; IFN-γ, interferon-γ; ILA, indole-3-lactic acid; OMVs, outer membrane vesicles).

Indole compounds produced by microbial metabolism, such as indole-3-carboxaldehyde (I3A), ILA, and indole-3-propionic acid (IPA), regulate immune responses primarily through the activation of the AhR (**Figure 3**). AhR is a ligand-activated transcription factor initially characterized as a receptor for environmental toxins that mediates dioxin toxicity (137). In recent years, AhR has been redefined as a pivotal molecular bridge linking environment-microbiota-host interactions and plays a central role in maintaining immune homeostasis (138). AhR expression in immune cells is both tissue-specific and cell-type-specific. By sensing diverse endogenous and exogenous ligands, it regulates the transcription of downstream target genes, thereby establishing it as a significant therapeutic target for autoimmune, inflammatory, and cancerous diseases (139). Within the tumor microenvironment, activation of the AhR signaling pathway profoundly impacts immune cell metabolism. For instance, TAMs in pancreatic ductal adenocarcinoma exhibit high AhR activity, which is associated with chronic exposure to indole derivatives. Furthermore, the expression level of the AhR in tissue samples frequently correlates with patients’ clinical outcomes, including disease progression and response to immunotherapy (140). Mechanistically, tryptophan metabolites regulate numerous metabolic pathways in immune cells via the AhR signaling axis. For example, AhR activation modulates the metabolic adaptability and inflammatory state of immune cells by regulating mitochondrial quality and function (141). AhR is also involved in regulating fatty acid uptake, synthesis, and oxidation in immune cells (142). Furthermore, AhR contributes to the temporal regulation of glycolysis (143).

Notably, AhR ligands from different sources can elicit strikingly divergent immunological outcomes. Metabolites from the kynurenine pathway, typically produced by tumor cells, often promote immunosuppression, whereas microbiota-derived indole derivatives can, in some contexts, enhance anti-tumor immunity. For example, Chang et al. demonstrated that inhibiting the AhR pathway blocks M2 polarization of TAMs, enhances their anti-tumor function, and thereby inhibits colorectal cancer growth (144). Conversely, I3A and ILA enhance intestinal barrier function via the AhR/IL-22 axis and promote the differentiation of inflammatory macrophages into homeostatic phenotypes, respectively, thereby alleviating colitis and delaying colon tumorigenesis (108, 145). Furthermore, indole-3-aldehyde mediates anti-tumor immunity by activating AhR in CD8^+^ T cells to promote IFN-γ production, and by promoting the ligand-dependent differentiation of CD4^+^ T cells into Th17 or Treg cells, ultimately enhancing the efficacy of immune checkpoint inhibitors (146). Notably, indole-3-acetic acid, derived from the gut microbe *Parabacteroides distasonis*, promotes phospholipid remodeling via the AhR-FASN axis, thereby increasing the sensitivity of bladder cancer cells to ferroptosis (147). This finding provides a novel perspective for combining metabolite-regulated tumor cell metabolism with immunotherapy. However, despite these advances, evidence directly linking microbiota-derived tryptophan metabolites to the regulation of immune cell metabolism and anti-tumor efficacy remains limited. Further studies are required to elucidate how AhR signaling precisely regulates metabolic enzyme activity, flux distribution, and energy transitions in immune cells.

### 4.3 Bile acids: the immune double-edged sword at the crossroads of metabolism and signal transduction

Bile acids are amphipathic molecules synthesized from cholesterol in the liver. Following metabolic modification by the intestinal microbiota, primary bile acids give rise to secondary bile acids, which act as important signaling molecules with complex roles in anti-tumor immunity. Primary bile acids, such as cholic acid (CA) and chenodeoxycholic acid (CDCA), undergo deconjugation by intestinal bacteria (e.g., *Bacteroides* and *Clostridium*) and are subsequently converted into secondary bile acids, primarily deoxycholic acid (DCA) and lithocholic acid (LCA). These bile acids regulate immune cell function by activating diverse receptors, including the farnesoid X receptor (FXR), vitamin D receptor (VDR), pregnane X receptor (PXR), and the G protein-coupled bile acid receptor 1 (TGR5/GPBAR1). The impact of secondary bile acids on CD8^+^ T cell function exhibits significant duality. For instance, DCA binds to receptors on CD8^+^ T cells, inhibits plasma membrane Ca^2+^-ATPase activity, blocks the Ca^2+^-NFAT signaling pathway, impairs glycolysis and effector functions, and ultimately promotes colorectal cancer proliferation and invasion (96). This finding reveals a mechanism whereby the intestinal microbiota promotes tumor immune escape via bile acid metabolism. Conversely, Zheng et al. demonstrated that secondary bile acids regulate CD8^+^ T cell energy metabolism and promote their functional activation via the G protein-coupled receptor TGR5, as well as through the mTOR and OXPHOS pathways (148). These seemingly contradictory results likely reflect the concentration dependence, receptor selectivity, and microenvironment specificity of bile acid signaling. Regarding innate immune regulation, bile acids are important regulators of macrophage polarization and function. For example, LCA inhibits hematopoietic stem cell activation by inducing apoptosis and suppressing transforming growth factor-β (TGF-β) signaling through both Smad-dependent and -independent pathways (149). Additionally, LCA inhibits glycolysis and promotes OXPHOS in macrophages, thereby promoting their differentiation into the M2 phenotype while suppressing polarization towards the M1 phenotype. This metabolic reprogramming provides a mechanistic basis for the role of bile acids in immune regulation.

A recent study demonstrated that microbiota-derived bile acids drive anti-tumor immunity by antagonizing the host androgen receptor (AR) (150). Specifically, bile acids bind to and inhibit the AR, thereby modulating tumor cell growth signaling pathways and activating immune cells to enhance anti-tumor immunity. *In vivo*, combination bile acid therapy enhanced the efficacy of anti-PD-1 treatment via this mechanism. This mechanism is notable because it expands the role of bile acids from purely metabolic regulation to the realm of endocrine-immune crosstalk, providing a novel perspective on microbe-host interactions.

### 4.4 Non-classical metabolites: mechanistic exploration of emerging immunometabolic regulators

Beyond traditional metabolites, numerous non-canonical metabolites produced by the microbiota are increasingly recognized for their immunomodulatory functions. Trimethylamine N-oxide (TMAO) is a microbial metabolite derived from choline, carnitine, and related compounds. In triple-negative breast cancer models, TMAO induces caspase-3-mediated cleavage of Gasdermin E (GSDME) by activating the endoplasmic reticulum stress sensor PERK, leading to tumor cell pyroptosis. This inflammatory cell death releases damage-associated molecular patterns (DAMPs) and tumor antigens, which potently activate DCs and recruit CD8^+^ T cells into the tumor, thereby enhancing anti-tumor immunity (151). This suggests that TMAO remodels the TME indirectly, by inducing immunogenic cell death (ICD), rather than by directly modulating immune cell metabolism. Phenylacetylglutamine (PAGln) is a metabolite derived from phenylalanine metabolism by intestinal flora. Elevated PAGln levels are negatively correlated with therapeutic responses to immune checkpoint inhibitors (ICIs) in cancer patients. Mechanistically, PAGln appears to inhibit glycolysis and OXPHOS in T cells via incompletely defined pathways, thereby impairing their metabolic adaptability and effector function and promoting immune escape (152). Thus, the specific molecular targets and signaling pathways of PAGln represent a major focus of current research.

## 5 Conclusion and prospect

Tumor immunometabolism has emerged as a crucial regulatory interface integrating the dynamics of the tumor microenvironment with the metabolic function of immune cells, representing a promising strategy to overcome the limitations of current immunotherapies. Microbial metabolites—collectively termed the host’s “second genome”—play a pivotal role in mediating this interaction. Key classes of these metabolites include SCFAs, bile acids, tryptophan derivatives, and other immunomodulatory compounds. Through the targeted modulation of fundamental metabolic pathways, including glycolysis, OXPHOS, and lipid metabolism, these microbial molecules exert precise control over immune cell survival, differentiation, and effector functions. SCFAs exemplify this functional versatility: butyrate, for instance, enhances FAO via CPT1A activation in induced Tregs (126, 128), yet also inhibits the STING pathway, which can reduce the efficacy of radiotherapy (82). Bile acids exert bidirectional effects on CD8^+^ T cell metabolism through TGR5 receptor signaling, either suppressing function by inhibiting the Ca^2+^-NFAT axis or promoting activation via mTOR stimulation (95). Similarly, tryptophan metabolites regulate mitochondrial homeostasis and function through AhR-dependent pathways (101, 147). These regulatory effects are highly contingent on metabolite concentration, identity, and the local conditions within the tumor microenvironment.

Despite considerable progress, clinical translation remains challenging due to substantial interindividual heterogeneity in gut microbiota composition, shaped by factors such as diet, age, geography, and comorbidities, as well as the complexity of mechanistic interactions among metabolites. To date, most studies have focused on single metabolites; however, the tumor microenvironment contains a complex mixture of metabolites whose synergistic or antagonistic effects remain poorly understood. Future research should prioritize unraveling the fine-grained regulatory networks governing microbial metabolite-immune cell metabolic crosstalk. Integrating multi-omics approaches—including metagenomics, metabolomics, and single-cell sequencing—will be key to constructing predictive models that link microbial signatures to immune metabolic activity and treatment response. Furthermore, developing innovative delivery systems, such as metabolite-loaded nanocarriers engineered to respond to TME conditions (e.g., low pH or hypoxia), represents a promising direction for precision therapeutic intervention.

In summary, microbial metabolites provide deep mechanistic insights and novel therapeutic opportunities for overcoming immunotherapy resistance. Advancing from descriptive studies to targeted metabolic interventions will be essential for ushering in a new era of personalized, combination cancer immunotherapy aimed at improving clinical outcomes and patient quality of life.
